# Clinical and genetic spectrum of 14 cases of NLRP3-associated autoinflammatory disease (NLRP3-AID) in China and a review of the literature

**DOI:** 10.1186/s13023-022-02364-z

**Published:** 2022-06-06

**Authors:** Yu Zhou, Wei Wang, Linqing Zhong, Lin Wang, Mingsheng Ma, Xiaoyan Tang, Zhuo Li, Changyan Wang, Lijuan Gou, Tiannan Zhang, Hongmei Song

**Affiliations:** 1 Department of Pediatrics, Peking Union Medical College Hospital, Chinese Academy of Medical Sciences, Beijing, China; 2 State Key Laboratory of Complex Severe and Rare Diseases, Peking Union Medical College Hospital, Beijing, China

**Keywords:** Inflammasomopathies, NLRP3, Autoinflammatory disease, NLRP3-AID

## Abstract

**Background:**

NLRP3-associated autoinflammatory disease (NLRP3-AID), caused by mutations of NLRP3, is one of the autoinflammatory diseases affecting inflammasomes. Since there are little cases of Chinese NLRP3-AID, we reported 14 Chinese NLRP3-AID patients in our center and summarized the clinical features of all Chinese patients by reviewing the literature.

**Results:**

Fourteen patients had been diagnosed as NLRP3-AID in our center. 12 different NLRP3 variants were identified, among which one is novel: p.Leu361Trp. Rash, recurrent fever, arthritis/arthralgia, uveitis, sensorineural deafness, symptoms of central neural systems (CNS), and increased inflammatory markers (including CRP, ESR, except Ferritin) were the common findings in Chinese patients. The frequencies of fever, neurological symptoms, musculoskeletal manifestations and ocular manifestations in Chinese patients might differ from that of patients from other regions. Besides, we also found clubbing fingers and optic neuritis in some NLRP3-AID patients, which were not commonly mentioned in previous reports.

**Conclusion:**

In our study, we expanded the clinical spectrum as well as the genetic pathogenic variants of NLRP3-AID. We also found that there were some differences between Chinese patients and patients from other regions, and that Chinese patients were more likely to develop severe symptoms.

**Supplementary Information:**

The online version contains supplementary material available at 10.1186/s13023-022-02364-z.

## Introduction

Systemic autoinflammatory diseases (SAIDs) are a growing group of disorders caused by a dysregulation of the innate immune system leading to episodes of systemic inflammation [1]. Most of them are caused by monogenic mutations. According to the 2019 International Union of Immunological Societies (IUIS) Classification of Primary Immunodeficiency Diseases, there are 45 kinds of autoinflammatory diseases, caused by 42 different mutated genes, and the number is still growing [2]. NLRP3-associated autoinflammatory disease (NLRP3-AID) is one of the first recognized autoinflammatory diseases, also recognized as Cryopyrin-associated periodic syndromes (CAPS) before. It encompasses a spectrum of autoinflammatory periodic fever syndromes with increasing severity: familial cold-associated syndrome (FCAS), Muckle-Wells syndrome (MWS), and neonatal-onset multisystem inflammatory disease (NOMID), also known as chronic infantile neurologic cutaneous articular syndrome (CINCA) [3]. In 1940s, FCAS was reported by Kile et al. [4], and the others, MWS and NOMID, were first described in 1962 and 1982 [5, 6]. Since then, plenty of cases series were reported, mostly in Caucasian population.

NLRP3-AID is caused by gain-of-function mutations of NLRP3. It leads to activation of caspase-1, which results in overexpression of proinflammatory cytokine IL-1β, and then brings various clinical manifestations, including recurrent episodes of fever associated with rash, arthritis/arthralgia, uveitis, sensorineural deafness, and symptoms of central neural system (CNS).

The prevalence of NLRP3-AID in USA is 1 in 1,000,000 people, while the incidence in France is about 1/360,000 [7, 8]. Such diseases were rarely reported in China, and many Chinese clinicians were not familiar to the manifestations of this disease, which leads to misdiagnosis or delayed diagnosis. Since NLRP3-AID has a highly broad spectrum of clinical manifestations, it is unknown whether Chinese patients present with some different or rare features. Here we reported 14 cases of NLRP3-AID in our medical center, reviewed all reports of Chinese NLRP3-AID cases, summarized the clinical features of them and compared with patients from other regions.

## Methods

### Patients

This study was performed at Peking Union Medical College Hospital (PUMCH) between January 2012 and December 2020. Standardized Case Report Form was used to record the demographic data, genetic results, and clinical presentations including symptoms, physical examinations, and laboratory results. Neurological symptoms and musculoskeletal symptoms could be further classified into two subgroups, mild or severe [9]. Morning headache, papilloedema, or aseptic meningitis is defined as mild neurological symptoms, whereas seizures, hydrocephalus, or mental retardation reflects severe neurological involvement. Mild musculoskeletal manifestations include myalgia and arthralgia, while patellar overgrowth, joint contractures, bone deformity, bone erosions, and osteolytic lesions are considered as severe manifestations. All patients were diagnosed as NLRP3-AID according to the combination of clinical manifestations and genetic results. Furthermore, FCAS is classified as presenting with fever and rash, without ocular manifestations or hearing loss; MWS is defined as urticaria-like rash not related to cold, predominant musculoskeletal manifestations, and sensorineural hearing loss; NOMID is diagnosed upon observing neonatal onset rash, chronic meningitis, and arthropathy with fever and inflammation are observed. This research was performed under the guide of the Declaration of Helsinki and approved by the Institutional Review Board of PUMCH (S-609, ZJS-1248, JS-1660).

### Detection of genetic variant

DNA samples were extracted from peripheral blood from patients and their parents. During 2012 to 2014, Sanger sequencing was directly applied to detect variants of NLRP3 (NM_004895). After 2014, we detected primary immunodeficiency diseases (PIDs) related genes according to IUIS classification by gene trapping high-throughput sequencing with the application of PIDs panel and then the variant locus of the patient and their parents would be verified by Sanger sequencing. Besides, the suspected variants would be further investigated in HGMD and OMIM database and the pathogenicity of the variants would be speculated by SIFT, Polyphen, MutationTaster, and GERP++. The pathogenicity of each variant is calculated according to ACMG criteria.

### Literature search

For English literature, we did a PubMed literature search for reports of Chinese NLRP3-AID between January, 2000 and December, 2020, with the indexing words: “NLRP3-associated autoinflammatory disease”, “Cryopyrin-associated periodic syndrome”, “familial cold autoinflammatory syndrome”, “Muckle-Wells syndrome”, “neonatal-onset multisystem inflammatory disease”, or “chronic infantile neurologic cutaneous articular syndrome”. For Chinese literature, similar searches were done in Wanfang database and CNKI database. Eight articles reporting Chinese cases of NLRP3-AID were found and we summarized the clinical presentations.

### Statistical analysis

Demographic results were expressed as median and range. Clinical presentations were described by numbers and rates of positive cases. Chi-square test or Fisher exact test was used for comparing frequencies of clinical manifestations between Chinese patients and patients from other countries by Statistical Product and Service Solutions (SPSS) 22.0 software; *P* < 0.05 would be considered as significant.

## Results

### Demographic data

By December 2020, 14 patients had been diagnosed as NLRP3-AID in our center. Demographic data are presented in Table 1.

**Table 1:** Demographic characteristics of 14 NLR-related autoinflammatory diseases patients

	n	%
Gender ratio (M/F)	10:4	
Positive Family history	2	14.3
	Median (IQR 25–75)	
Age at onset (month)	0 (0–6)	
Age at first visit (month)	22.5 (7.5–76.8)	
Age at diagnosis (month)	54.5 (17.5–110.8)	

All patients had disease-onset during childhood, and the Male-to-Female ratio in this case series is 10:4. Most patients had disease onset within 1 month old, and the onset age ranges from right after birth to about 5 years old (IQR 25–75 0–6 m). The median age of first visit for medical service is 22.5 months, while the median age of diagnosis is 54.5 months.

### Gene variants

**Fig. 1:**
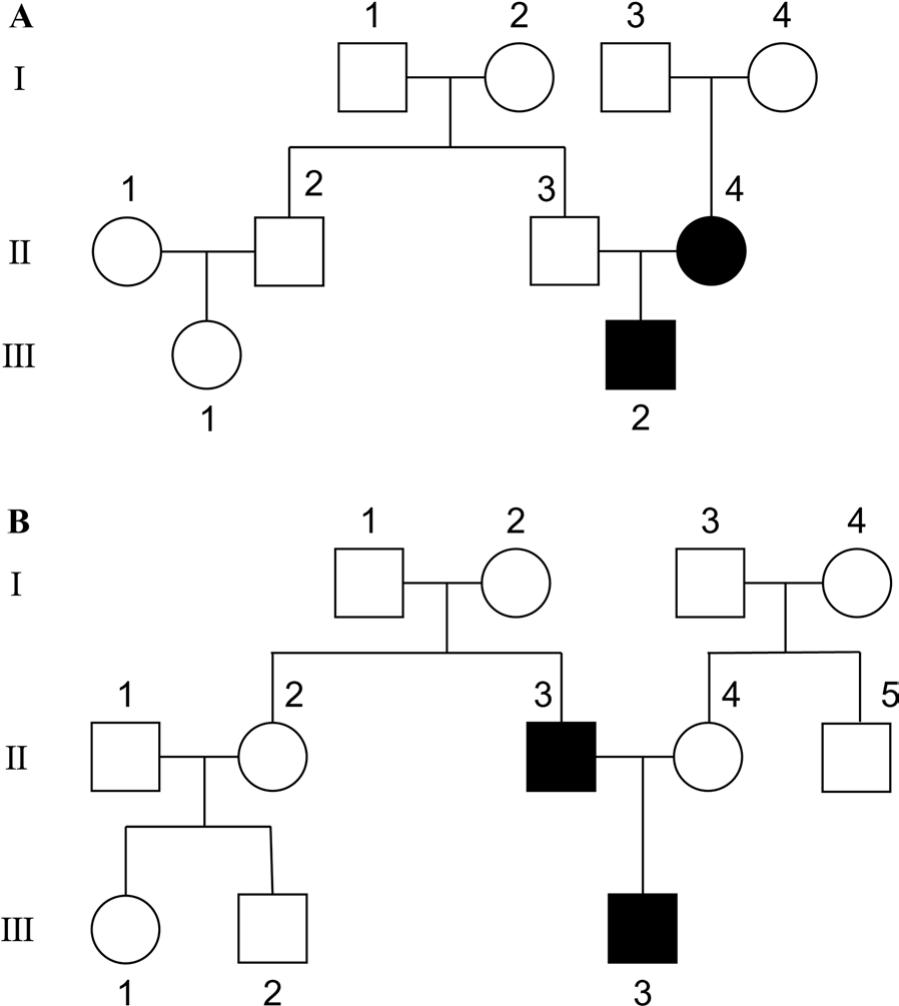
The family trees of the 2 familial forms NLRP3-AID patients carrying c.1049C > T, p.Thr350Met (**A**) and c.983G > A, p.Gly328Glu (**B**). III-2 (**A**) and III-3 (**B**) refer to the probands

We found 12 NLRP3 variants which cause 11 different amino acid substitutions in NLRP3 in these patients and listed them in Table 2, and the electropherogram for the variants was shown in Additional file 1: Fig S1. Among those variants, one is novel: c.1082T > G, p.Leu361Trp. Other variants were reported in previous studies and could be found in https://infevers.umai-montpellier.fr/web/. In terms of the p.Leu361Trp variant, it has 0 frequency in several databases like 1000g2015aug_all, ESP6500si, ExAC_ALL, ExAC_EAS, as well as mbiobank (http://www.mbiobank.com), a database for Chinese population. Although it is expected to be Benign in Mutation Taster, the speculated pathogenicity is “Damaging” (SIFT), or “Probably damaging” (PolyPhen). Combing the genetic analyzed data and clinical feature of P14, we believe that mutated allele is pathogenic, although functional studies have not been finished yet. Besides, two patients inherited the pathogenic alleles from one of their parents who were also patients of NLRP3-AID. The family trees were shown in Fig. 1.

**Table 2:** Genetic results of 14 NLR-related autoinflammatory diseases patients

	Diagnosis	Gene	Variant†	Pathogenicity	Inheritance	Family history
1	FACS	NLRP3	c.932T > C, p.Phe311Ser	Pathogenic	De novo	No
2	FACS	NLRP3	c.796C > T, p.Leu266Phe	Pathogenic	De novo	No
3	FACS	NLRP3	c.1049C > T, p.Thr350Met	Pathogenic	Maternal	Yes
4	MWS	NLRP3	c.1311G > T, p.Lys437Asn	Pathogenic	De novo	No
5	MWS	NLRP3	c.1049C > T, p.Thr350Met	Pathogenic	De novo	No
6	MWS	NLRP3	c.1711G > A, p.Gly571Arg	Pathogenic	De novo	No
7	NOMID	NLRP3	c.1715A > G, p.Tyr572Cys	Pathogenic	De novo	No
8	NOMID	NLRP3	c.1711G > C, p.Gly571Arg	Pathogenic	De novo	No
9	NOMID	NLRP3	c.1991T > C, p.Met664Thr	Pathogenic	De novo	No
10	NOMID	NLRP3	c.1991T > C, p.Met664Thr	Pathogenic	De novo	No
11	NOMID	NLRP3	c.983G > A, p.Gly328Glu	Pathogenic	Paternal	Yes
12	NOMID	NLRP3	c.913G > A, p.Asp305Asn	Pathogenic	De novo	No
13	NOMID	NLRP3	c.918G > T, p.Glu306Asp	Likely pathogenic	De novo	No
14	NOMID	NLRP3	c.1082T > G, p.Leu361Trp*	Likely pathogenic	De novo	No

*Represents novel variant;

^†^The reference sequence is NM_004895

### Clinical presentations

**Fig. 2:**
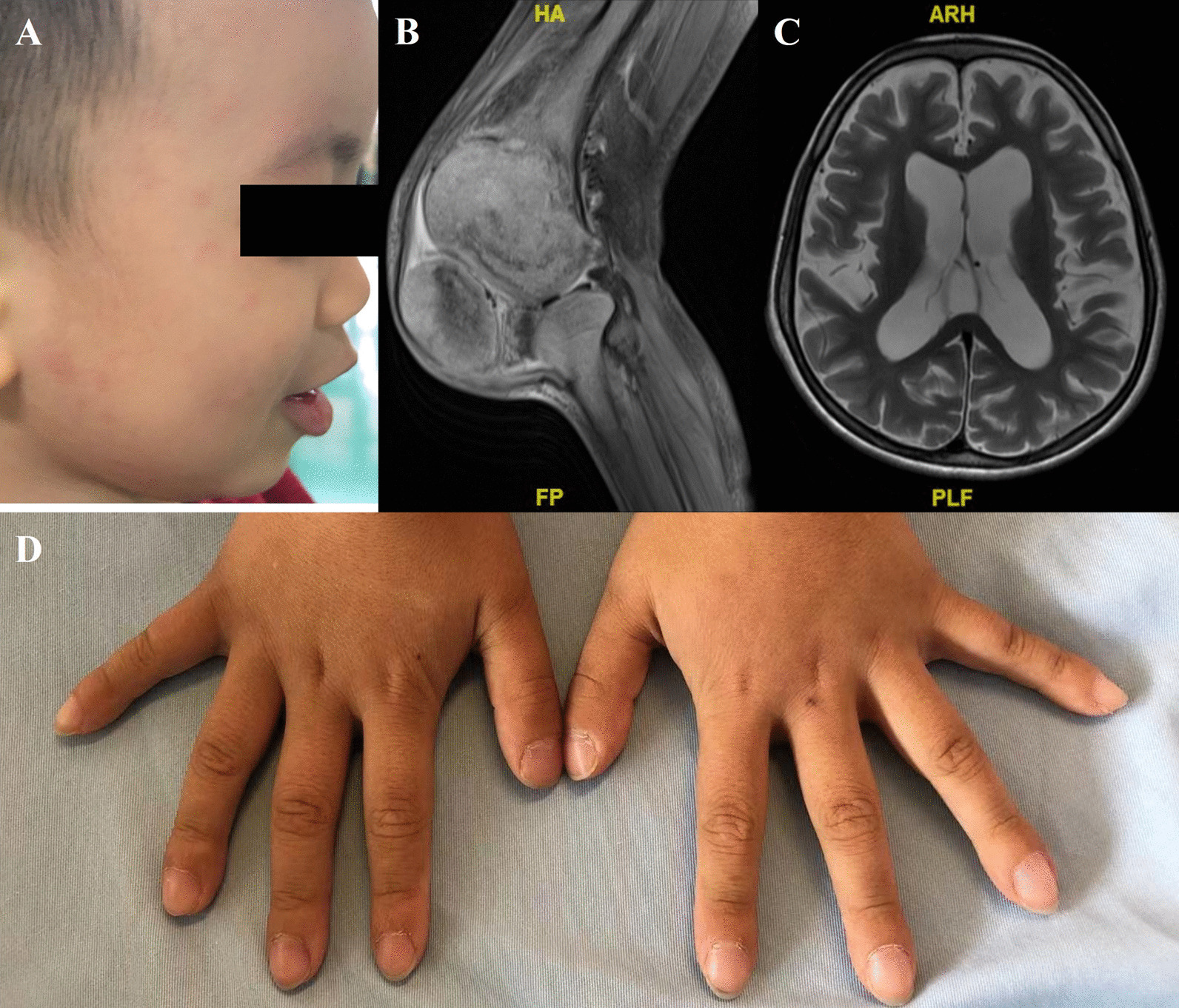
Rash (**A**), joint deformation (**B**), neurological involvement (**C**) and clubbing fingers (**D**) of NLRP3-AID patients

All patients in our cohort developed rash during acute phase. Among them, urticaria-like rash were seen in 8 patients (P2, P3, P7, P9, P10, P11, P12, and P14), while macules or papules were found in other patients. Fever was also observed in most patients (13/14). The duration of fever was about 1 to 10 days, while the interval between flares was usually around 2 weeks to 1 month. Besides, 12 patients had neurological involvements, and most of them had mild neurological symptoms like headache and aseptic meningitis. There were 5 patients having severe neurological involvement. Among them, 3 patients (P4, P9, and P10) presented with mental retardation, 3 patients (P4, P9, and P13) had hydrocephalus, and 3 patients (P4, P8, and P9) developed encephalatrophy. Another common characteristic of NLRP3-AID is musculoskeletal involvement (8/14). Mild musculoskeletal symptoms, such as myalgia or arthralgia/arthritis were common, while 4 patients (P4, P7, P9, and P13) showed severe musculoskeletal symptoms such as joint deformity, frontal bossing, or bone erosions indicated by radiographic examinations like X-ray or MRI. A detailed summary of the clinical manifestations is shown in Table 3. Hearing loss and ocular manifestations were also common in NLRP3-AID, and the percentages were 42.9% (6/14) and 28.6% (4/14), respectively. There were two patients with clubbing fingers (P9 and P12, Fig. 2) and another one patient (P5) developing optic neuritis, both of which were uncommon manifestations of NLRP3-AID in previous reports.

**Table 3:** Clinical manifestations of 14 NLR-related autoinflammatory diseases patients

Patient No	Fever	Rash	Ocular symptoms	Hearing loss	Neurological symptoms	Severe neurological symptoms	Musculoskeletal manifestations	Severe Musculoskeletal manifestations	Growth retardation	Leukocytosis	Anemia	Thrombocytosis	Increased CRP/ESR	Increased Ferritin	ANA (+)	Proteinuria
1	√	√			√					√		√	√	NA		
2	√	√			√				√	√	√	√	√	NA		
3	√	√								√			√	NEG		
4	√	√			√	√	√	√	√	√		√	√	√	√	√
5	√	√	√	√	√		√			√		√	√	NA		
6	√	√		√						√		√	√	NA		
7		√		√	√		√	√		√	√	√	√	NEG		√
8	√	√			√	√				√	√	√	√	NA		
9	√	√	√	√	√	√	√	√	√	√	√	√	√	NEG		
10	√	√			√	√			√	√	√	√	√	NEG		
11	√	√			√		√			√	√	√	√	NEG		
12	√	√	√	√	√		√		√	√	√	√	√	NEG		
13	√	√		√	√	√	√	√		√	√	√	√	NEG		
14	√	√	√		√		√			√	√	√	√	NEG		

*NA* Test was not performed, *NEG* negative

In terms of laboratory examinations, highly increased acute phase reactants (APRs) including CRP and ESR were still sensitive to identify the potential patients of NLRP3-AID, since increased APRs were observed in all patients during the attacks. However, different from other inflammatory diseases such as systemic Juvenile Idiopathic Arthritis (sJIA), increased Ferritin was uncommon in NLRP3-AID (1/9). Leukocytosis and thrombocytosis were also observed in most patients due to the active inflammation status. In the meantime, anemia was mostly found in patients diagnosed as NOMID. In terms of autoimmune antibodies, only one patient (P4) showed low titer of ANA (1:100), while most patients had negative antibodies as in other autoinflammatory diseases. Besides, compared to autoimmune diseases, renal involvement was much less common in NLRP3-AID. In our center, there were only two patients (P4 and P7) suspected to have mild proteinuria because of Protein ( +) in urine routine test, and both had 24 h urine protein less than 0.5 g.

For treatment, the first-line therapy for NLRP3-AID is to block the pathway of IL-1β, including anakinra (recombinant IL-1 receptor antagonist), rilonacept (IL-1β trap) and canakinumab (monoclonal antibody against IL-1β) [10]. However, none of them was available in the mainland of China. In our clinical practice, glucocorticoid treatment could reduce the frequency of attacks and decrease APRs. However, glucocorticoid alone could not stop the progression of diseases. Since most patients had increased IL-6, some patients were treated with Tocilizumab, which successfully controlled the fever symptom. Of note, the long-term responses are still unclear, and more follow-up data is required.

### Literature review and comparison

In 2014, PRINTO and Eurofever summarized the phenotypic and genotypic characteristics of NLRP3-AID, based on the results of the largest NLRP3-AID cohort, consisting of 136 patients [9]. In East Asian area, Japan is the earliest to report NLRP3-AID in 1976 and did a review of all Japanese NLRP3-AID patients in 2012 [11]. However, there was no such report in China until Li et al. reported 15 cases of NLRP3-AID in Chinese population in 2017 [12]. Up to now, there were only eight articles, describing 28 Chinese NLRP3-AID patients (Additional file 2: Table S1) [12–19]. Here, after combining data from our center, we compared the clinical manifestations among Chinese, Japanese, and western patients (Table 4). Fever (41/42, 97.6%) and rash (39/42, 92.9%) were the major symptoms in Chinese population, which was the same as previous reported in other regions. However, the frequency of fever in Chinese population is much higher than that in Japanese (14/19, 73.7%, *p* = 0.009) or western population (108/136, 79%, *p* = 0.005). Unlike western patients, Chinese patients had a higher frequency of neurological involvement (21/35, 60%), as well as severe neurological manifestations (6/20, 30%) (*p* = 0.038 and 0.04). While about 60%-70% NLRP3-AID patients had ocular manifestations in previous reports, only 35.7% Chinese patients suffered from it (*p* = 0.046, and < 0.001, respectively). Besides, the percentage of musculoskeletal manifestations seemed lower in East Asian population (66.7% in China and 68.4% in Japan vs 86% in Caucasia, *p* = 0.005). Note that, although less patients showed musculoskeletal manifestations, compared with western patients, Chinese patients seem to exhibit higher frequencies of severe symptoms (*p* = 0.025).

**Table 4:** Comparison of clinical manifestations of CAPS among Chinese patients, Japanese patients, and western patients

	Total	Japan	p1 value	Western	p2 value
*n*	42	19		136	
Origin	Chinese	Japanese		Most Caucasian	
Gender Ratio	26:16	11:8	0.767^a^	69:67	0.205^a^
Fever	41 (97.6%)	14 (73.7%)	0.009^b^	108 (79%)	0.005^a^
Rash	39 (92.9%)	19 (100%)	0.545^b^	132 (97%)	0.358^b^
Ocular manifestations	15 (35.7%)	12 (63.2%)	0.046^a^	97 (71%)	< 0.001^a^
Hearing loss	15 (35.7%)	7 (36.8%)	0.932^a^	56 (41%)	0.527^a^
Neurological symptoms	21/35 (60%)^†^	NA		55 (40%)	0.038^a^
Severe Neurological symptoms	6/20 (30%)^‡^	NA		16 (12%)	0.040^b^
Musculoskeletal manifestations	28 (66.7%)	13 (68.4%)	0.892^a^	117 (86%)	0.005^a^
Severe Musculoskeletal manifestations	4/20 (20.0%)^‡^	NA		6 (4%)	0.025^b^

^a^Compared by Chi-square test, ^b^Compared by Fisher exact test

^†^Reference [13] didn’t describe the CNS symptoms and thus 7 patients in their cohort were removed in the comparison of neurological symptoms

^‡^References [12, 13] didn’t describe the detailed manifestations of CNS and musculoskeletal system, and thus patients in their cohort (15 and 7, respectively) were removed when comparison was performed in terms of severe symptoms

p1 refers Chinese population versus Japanese population, p2 refers Chinese population versus western population

## Discussion

Our study detailly summarized the clinical and genotype features of Chinese NLRP3-AID patients. our data consisted with previous clinical reports in many respects. Fever and rash were the most prevalent clinical presentations while chronic meningitis and neurosensory hearing loss were frequently seen in NLRP3-AID patients. Unlike autoimmune diseases such as systemic lupus erythematosus, the renal involvement was rare in NLRP3-AID. Some of our patients were once misdiagnosed as sJIA for fever and arthritis, and we found that most NLRP3-AID patients didn’t show increased Ferritin, which is usually high in sJIA, and it could be a clue to help clinicians to distinguish those diseases. When compared with patients from other regions, Chinese patients were less likely to suffer from musculoskeletal manifestations as well as ocular manifestations. However, the frequency of severe symptoms, either neurological or musculoskeletal, was higher in Chinese populations, indicating a more progressive process in Chinese patients. It also highlights the importance of early diagnosis and treatment. Meanwhile, the lack of reports about Chinese NLRP3-AID patients leads to poor recognition of this disease in China. Resonating with this fact, the time from disease onset to final diagnosis in our cohort could be as long as 18 years. And the delay may also contribute to more severe symptoms.

Furthermore, we also found some rare presentations in our patients. We found clubbing fingers in two of our patients, though they did not show symptoms of dyspnea and their pulmonary function and CT were both normal. A possible explanation might be that the activation of NLRP3 inflammasome lead to the overproduction of vascular endothelial growth factor (VEGF), and platelet-derived growth factor (PDGF) [20]. And those growth factors are proved to incite changes of clubbing [21]. Another rare manifestation in our cohort is optic neuritis, which happened on P5 during follow-up period. The clinical presentation was rapid loss of sight on one side, and MRI showed line-like enhancement of the intraorbital segment of optic nerve and the edge of optic chiasm, suggesting optic neuritis. After treating with high dose of intravenous methylprednisolone, his MRI gradually returned normal. However, in terms of vision, no significant improvement was observed. It revealed another possible disabling outcome of NLRP3-AID and alert physicians to pay more attention to ocular symptoms.

In terms of the association of phenotypes and variants, Levy et al. pointed out that Thr350Met was associated with hearing loss while Asp305Asn was linked to severe phenotype [9]. In our cohort, we also noticed that P5 carrying Thr350Met, developed aseptic meningitis and sensorineural hearing loss, and thus was diagnosed as MWS. And P12 who carried Asp305Asn, was diagnosed as NOMID. Both were consistent with the previous findings. Sometimes the same variant can also cause different clinical phenotypes. Of note, in our cohort, p.Gly571Arg was found in both MWS (P6) and NOMID (P8) patients, and p.Thr350Met was found in both FACS (P3) and MWS (P5), suggesting that the phenotype is not only determined by mutated allele of NLRP3, but is also influenced by other factors. Another interesting example is P11 and his father. P11 inherited p.Gly328Glu from his father and was diagnosed as NOMID because of early onset fever, urticaria-like rash, and neurosensory involvement. However, it seemed that his father was a MWS patient as he occasionally had rash or arthralgia since his childhood and developed hearing loss at the age of 16. It is rare that patients carrying the same variant in the same family should have different diagnosis of NLRP3-AID subtypes.

In addition, we also report 1 novel variation of NLRP3 which might be pathogenic for NLRP3-AID. However, functional tests haven’t been finished yet, which is a limitation. Another limitation in our research is that it is a single center research, and it might not be enough to represent the whole Chinese NLRP3-AID patients, although we summarized all Chinese cases by reviewing relevant literatures. A nationwide multi-center registry study is needed in the future.

## Conclusion

To summary, the main clinical manifestations of NLRP3-AID in Chinese patients consist of fever, rash, CNS symptoms and musculoskeletal symptoms. The percentages of fever, neurological symptoms as well as severe neurological symptoms, and severe musculoskeletal symptoms in Chinese patients are higher than in western patients, while ocular manifestations and musculoskeletal symptoms are less common. We also report that clubbing fingers and optic neuritis can be found in NLRP3-AID patients and expand the clinical spectrum of NLRP3-AID. Besides, we report one novel pathogenic variant causing NLRP3-AID.

## Supplementary Information

**Additional file 1: Figure S1.** NLRP3 gene variations in our cohort. Of note, P2, P11 and P14 received genetic tests in other hospitals and refused reexamination in our center.

**Additional file 2: Table S1.** Detailed clinical manifestations of CAPS patients in China according to literature.

## Data Availability

Please contact author for data requests.
