# Supplementary figures and images for: Clinical and genetic spectrum of 14 cases of NLRP3-associated autoinflammatory disease (NLRP3-AID) in China and a review of the literature
Source: Orphanet J Rare Dis. 2022 Jun 6;17:214. doi: 10.1186/s13023-022-02364-z (PMC9169254; doi:10.1186/s13023-022-02364-z)

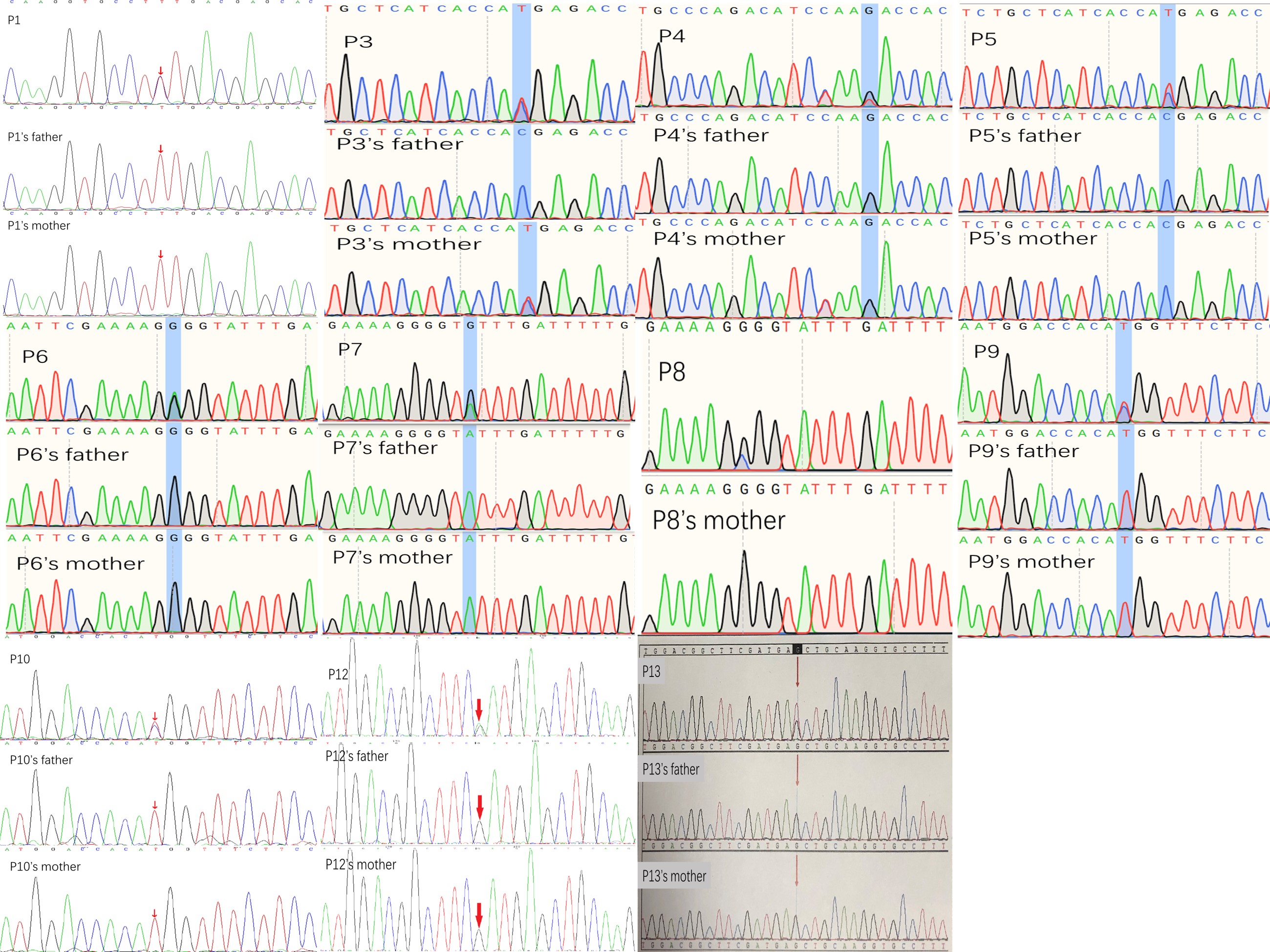

Supplement: Supplementary file 1 — Additional file 1: Figure S1. NLRP3 gene variations in our cohort. Of note, P2, P11 and P14 received genetic tests in other hospitals and refused reexamination in our center. [file 13023_2022_2364_MOESM1_ESM.jpg]
