# Supplementary material for: Clinical and genetic spectrum of 14 cases of NLRP3-associated autoinflammatory disease (NLRP3-AID) in China and a review of the literature
Source: Orphanet J Rare Dis. 2022 Jun 6;17:214. doi: 10.1186/s13023-022-02364-z (PMC9169254; doi:10.1186/s13023-022-02364-z)
Supplement: Supplementary file 2 — Additional file 2: Table S1. Detailed clinical manifestations of CAPS patients in China according to literature. [file 13023_2022_2364_MOESM2_ESM.docx]

Table S1. Detailed clinical manifestations of CAPS patients in China according to literature

|  | Our center | Ref 12 | Ref 13 | Ref 14 | Ref 15 | Ref 16 | Ref 17 | Ref 18 | Ref 19 | Total |
| --- | --- | --- | --- | --- | --- | --- | --- | --- | --- | --- |
| *n* | 14 | 15 | 7 | 1 | 1 | 1 | 1 | 1 | 1 | 43 |
| Origin | Chinese | Chinese | Chinese | Chinese | Chinese | Chinese | Chinese | Chinese | Chinese | Chinese |
| Gender Ratio | 10:4 | 8:7 | 5:2 | M | F | M | F | M | F | 26:17 |
| Fever | 13 (92.9%) | 15 (100%) | 7 (100%) | 1 | 1 | 1 | 1 | 1 | 1 | 42/43 (97.7%) |
| Rash | 14 (100%) | 14 (93.3%) | 4 (57%) | 1 | 1 | 1 | 1 | 1 | 1 | 39/43 (90.7%) |
| Ocular manifestations | 4 (28.6%) | 7 (46.7%) | 2 (29%) | 1 | 0 | 0 | 0 | 1 | 0 | 15/43 (34.9%) |
| Hearing loss | 6 (42.9%) | 5 (33%) | 2 (29%) | 1 | 0 | 0 | 1 | 0 | 0 | 15/43 (34.9%) |
| Neurological symptoms | 12 (85.7%) | 7 (46.7%) | NA | 0 | 0 | 0 | 1 | 0 | 1 | 21/36 (58.3%) |
| Severe | 5 (35.7%) | NA | NA | 0 | 0 | 0 | 0 | 0 | 1 | 6/21 (28.6%) |
| Musculoskeletal manifestations | 8 (57.1%) | 11 (73.3%) | 5 (71%) | 0 | 1 | 1 | 1 | 1 | 0 | 29/43 (67.4%) |
| Severe | 4 (28.6%) | NA | NA | 0 | 0 | 0 | 0 | 0 | 0 | 4/21 (19.0%) |
| Increased CRP/ESR | 14 (100%) | 15 (100%) | NA | 1 | 1 | 1 | 1 | 1 | 1 | 36/36 (100%) |
